# Vitamin D deficiency and associated factors in south Korean childbearing women: a cross-sectional study

**DOI:** 10.1186/s12912-021-00737-6

**Published:** 2021-11-01

**Authors:** Yanghee Pang, Oksoo Kim, Jung-Ah Choi, Heeja Jung, Jui Kim, Haeok Lee, Hyangkyu Lee

**Affiliations:** 1 College of Nursing, Ewha Womans University, Seoul, South Korea; 2 Ewha Research Institute of Nursing Science, Seoul, South Korea; 3 College of Nursing, Konyang University, Daejeon, South Korea; 4 College of Nursing, Yonsei University, Seoul, South Korea; 5 Strategy and Planning, Samsung Medical Center, Seoul, South Korea; 6 Department of Nursing, Ansan University, Gyeonggi-do, Korea; 7 College of Nursing and Health Science, University of Massachusetts Boston, Boston, MA USA; 8 Mo-Im Kim Nursing Research Institute, Yonsei University, Seoul, South Korea

**Keywords:** 25-hydroxyvitamin D, Vitamin D deficiency, Childbearing age

## Abstract

**Background:**

Adequate levels of vitamin D are important for women of childbearing age as vitamin D helps maintain the pregnancy and ensures proper maternal and fetal bone metabolism and fetal skeletal development. However, vitamin D deficiency is a health problem prevalent in women of all ages, worldwide. This study aimed to determine the current status of serum vitamin D levels and the risk factors for vitamin D deficiency among South Korean nurses of childbearing age.

**Methods:**

Serum levels of 25-hydroxyvitamin D (25[OH]D) were measured in 1594 registered nurses aged 20 to 45 years who are participants in an ongoing prospective cohort study of the Korean Nurses’ Health Study initiated in 2013. The participants completed surveys about demographic and occupational characteristics and physical and psychological health. We examined associations with vitamin D deficiency through multivariable logistic regression analysis.

**Results:**

The average blood 25(OH) D concentration of the participants was 12.92 ng/mL (4.0–63.4 ng/mL), while the prevalence of vitamin D deficiency (< 20 ng/mL) was 89% (1419/1594). Multivariable logistic regression showed that significant risk factors for vitamin D deficiency included month of sampling (there was a lower level of vitamin D deficiency in winter than in spring, summer, or fall), age (women in their 20s had a lower vitamin D level than those in their 30s and 40s), and stress symptoms. Vitamin D levels were not associated with body mass index, physical activity, and depressive symptoms.

**Conclusions:**

This study showed a high prevalence of vitamin D deficiency in Korean female nurses. Serum levels of vitamin D were associated with age and season. Vitamin D deficiency should be recognized as one of the primary health concerns among young women. More proactive actions, such as vitamin D supplements and food fortification, are needed to improve vitamin D deficiency in high-risk groups.

## Background

Vitamin D deficiency is very prevalent worldwide, though the prevalence varies according to age, region, culture, and lifestyle [1]. It has become a global health issue requiring prioritization in prevention and treatment because it is related to the development of musculoskeletal disorders, such as osteoporosis and osteopenia, as well as chronic diseases, such as cardiovascular disease, diabetes, and cancer [2]. According to a national study, 71.7% of the population in South Korea over the age of 10 had a serum 25-hydroxyvitamin D (25[OH]D) concentration of less than 20 ng/mL [3], showing a higher prevalence of vitamin D deficiency than the populations of China and Australia. In China, 46% of females and 30% of males over the age of 20 have inadequate 25(OH) D levels [4], while in Australia, 30% of adults over the age of the 25 have a vitamin D deficiency [5].

Pregnancy, obesity, reduced exposure to sunlight, and being a person of color are all factors that can increase the risk of vitamin D deficiency. In pregnant women, low serum vitamin D levels have been associated with gestational diabetes, preterm birth, and poor health outcomes for premature newborns [6]. In South Korea, vitamin D deficiency is reported more frequently in females who are younger than 30 years old rather than in older females [7, 8]. Therefore, it is vital to determine the factors related to vitamin D deficiency in younger females.

Vitamin D is primarily synthesized in the skin during exposure to sunlight [9]. The month in which blood is collected for testing and the level of outdoor physical activity are factors that most influence vitamin D levels [10, 11]. Other factors, including working conditions (e.g., working indoors or being a shift worker) and working longer than 40 h, are reported to increase the risk of vitamin D deficiency [12, 13].

Recent studies that examined the relationship between vitamin D levels and mental health have found that low 25(OH) D levels caused a pro-inflammatory state and could be associated with depression [14]. Studies using longitudinal data also reported that the level of vitamin D at baseline and the incidence of depression showed a statistically significant relationship [15]. Furthermore, study participants with high vitamin D levels had positive self-perceived mental health and low stress levels [16], which has led to the widespread belief that psychological health is related to vitamin D levels.

As demonstrated in these previous studies, inadequate vitamin D levels are related to many factors, including sunlight exposure, ethnicity, gender, indoor and shift work, and physical and mental health. Considering the high prevalence of vitamin D deficiency in South Korean women and its negative impact on women’s health as well as the high rate of chronic diseases in this population, low serum 25(OH) D levels in women of childbearing age require attention. However, previous studies primarily investigated the commonalities of vitamin D deficiencies in male and female adults and seniors. Therefore, studies to identify specific characteristics related to low vitamin D levels in women of childbearing age are crucial. In this study, we focused on the impact of working patterns and health characteristics on serum vitamin D levels in women of childbearing age.

## Methods

### Study design and participants

This study was designed as a cross-sectional study that analyzed data from the Korean Nurses’ Health Study (KNHS) [17]. The KNHS is the first large-scale prospective longitudinal study in South Korea conducted on 24–45-year-old female nurses with more than one year of nursing experience. The KNHS survey is based on the study protocol and questions used in the Nurses’ Health Study (NHS) of the United States [18]. The baseline survey (Module 1) began in 2013 with 20,613 female nurses, and follow-up surveys were conducted. Details on demographic characteristics, anthropometric measures, health screening, illnesses, family history, health behavior, lifestyle and diet, reproductive health, pregnancy, mood and subjective health perception, employment, and occupational exposure were collected. Module 5 was conducted between November 2016 and September 2017, and blood samples were collected from 2000 participants. For this study, we analyzed the data from 1594 participants who had completed both the Module 3 and Module 5 survey questions and had recorded serum vitamin D levels [19].

### Data collection

In order to recruit survey participants for Modules 3 and 5, information on study participation was sent using the mobile phone numbers and email addresses captured at baseline. Volunteers participated in the survey by accessing the KNHS website (either online or via the mobile phone). The top 30 hospitals were chosen based on the number of participants at baseline. Of those, 12 hospitals cooperated and indicated their availability. These were selected as sites for blood testing. Promotional materials were posted at each of the 12 hospitals to recruit more participants from the baseline data group for blood testing. In addition, email and text messages with information on the blood test were sent out to all eligible participants to request voluntary participation.

After applications were completed by the nurses who had volunteered to participate, instructions on how to prepare for the blood draw, including fasting beforehand, were delivered to participants in collaboration with the nursing department of each hospital. A research team comprising 5–6 members (trained nurses, support staff, and phlebotomists) visited the designated hospital for 3–10 days. The phlebotomist drew blood samples by venipuncture of the median antecubital vein using a vacuum system in volunteers who had fasted for 8 h. The collected blood was stored and transported according to the process followed by certified laboratories (Green Cross LabCell, South Korea). The serum 25(OH) D level was analyzed using an autoanalyzer (Cobas 8000, Roche, Switzerland).

The 25(OH) D levels were classified as deficient or non-deficient using a 25(OH) D concentration of 20 ng/mL as a cutoff [20]. The months of the blood draw were grouped based on the season: spring (March–May), summer (June–August), fall (September–November), and winter (December–February).

Further, the survey questioned participants on their weekly average of overtime hours and whether they worked on a shift. In this study, shift work was defined as “day shift and evening shift and/or night shift in a sequential basis.”

The height and weight of participants reported in Module 5 were used to calculate the participant’s body mass index, which is calculated by dividing the weight (kg) by the height in meters squared (m^2^). In order to determine the level of physical activity, the participants were asked how much of each of the following activities they performed on average over the past year: walking, jogging, running, cycling, tennis/squash/racquetball, lap swimming, dancing, skiing, aerobic exercise (e.g., using a stepper), yoga, simple workouts (e.g., stretching and calisthenics), and intense physical activities (e.g., weight training). Metabolic equivalent of task (MET) values (hours/week) for each physical activity were calculated using the criteria suggested by previous studies [21].

Depressive symptoms were measured using the Patient Health Questionnaire (PHQ-9) [22] which is composed of 9 questions with a possible score ranging from 0 to 27, with higher scores indicating more severe depression. In this study, the depressive symptoms were divided into 5 levels, namely: minimal (0–4), mild (5–9), moderate (10–14), moderately severe (15–19), and severe (more than 20). The Cronbach’s alpha reliability estimate in the present study was 0.86, which is similar to the Cronbach’s alpha reliability estimate of 0.87 in the original PHQ-9 study [23].

The South Korean version of the Perceived Stress Scale-10 [24], composed of 10 questions, was used to measure the level of stress, with a possible score ranging from 0 to 40 points. The total scores for the stress questions were used. Questions 4, 5, 7, and 8 were reverse coded, i.e. higher scores indicated a higher level of stress. The Cronbach’s alpha reliability estimate in the present study was 0.70, and that of the original study was 0.819 [24].

### Statistical analysis

SPSS version 23 (SPSS Inc., Chicago, IL, USA) was used to analyze data. Data were expressed as frequencies and percentages or averages and standard deviations following a descriptive statistical analysis. In contrast, the chi-square test and t-test were used to analyze the differences between the participants who had been classified as having deficient and non-deficient vitamin D levels. These were further grouped by general and occupational characteristics and physical and psychological health. The statistical significance of vitamin D deficiency was confirmed using a multivariable logistic regression analysis. The timing of blood tests, demographic factors, work environment factors, and physical and psychological factors were input into Models 1–4.

## Results

### Participant characteristics

The mean concentration of serum 25(OH) D was 12.92 ng/mL, ranging from 4 to 63.4 ng/mL A total of 1419 participants (89%) had a vitamin D concentration of < 20 ng/mL, and 175 participants (11%) had vitamin D levels of ≥ 20 ng/mL. There were 1127 (70.7%) shift workers, with an average of 5.26 (SD = 4.71) overtime working hours. The chi-square tests indicated that the levels of vitamin D level differed significantly based on the sampling month, age, marital status, education, and shift work (Table 1).

**Table 1:** Vitamin D levels according to baseline characteristics (*N* = 1594)

Variable		Total	Vitamin D level		χ^2^ / t
		N(%) or M ( SD)	< 20 ng/mL (*N* = 1419)	20 ng/mL ≤ (*N* = 175)	
Sampling month	12–2	575(36.1)	537(37.8)	38(21.7)	17.599^**^
	3–5	235(14.7)	203(14.3)	32(18.3)	
	6–8	571(35.8)	495(34.9)	76(43.4)	
	9–11	213(13.4)	184(13.0)	29(16.6)	
Age (years)		33.15(6.32)	32.89 ( 6.28)	35.26 ( 6.24)	−4.716^***^
	20–29	593(37.2)	558(39.3)	35(20.0)	25.486^***^
	30–39	697(43.7)	603(42.5)	94(53.7)	
	40≤	304(19.1)	258(18.2)	46(26.3)	
Marital status	Unmarried	850(53.3)	776(54.7)	74(42.3)	9.625^**^
	Married or other	744(46.7)	643(45.3)	101(57.7)	
Education	Diploma	312(19.6)	290(20.4)	22(12.6)	8.228^*^
	Bachelor	1003(62.9)	890(62.7)	113(64.6)	
	Master’s or higher	279(17.5)	239(16.8)	40(22.9)	
Shift work	Yes	1127(70.7)	1019(71.8)	108(61.7)	7.667^**^
	No	467(29.3)	400(28.2)	67(38.3)	
Overtime hours (week)		5.26 ( 4.71)	5.33 ( 4.76)	4. 66(4.32)	1.778

^*^p < .05, ^**^p < .01, ^***^p < .001.

With regard to physical and psychological traits, the physical activity level of the participants was 22.34 (33.22 ) METs (hours/week). Further, 750 (47.1%) participants exhibited minimal levels of depressive symptoms. The stress symptoms scores showed a significant difference based on vitamin D levels (Table 2).

**Table 2:** Vitamin D level according to physical and psychological characteristics (*N* = 1594)

Variable		Total	Vitamin D level		χ^2^ or t
		N(%) or M ( SD)	< 20 ng/mL (*N* = 1419)	20 ng/mL ≤ (*N* = 175)	
BMI	Underweight	191(12.0)	170(12.0)	21(12.0)	1.074
	Normal	980(61.5)	878(61.9)	102(58.3)	
	Overweight	423(26.5)	371(26.1)	52(9.7)	
Physical activity		22.34 ( 33.22)	22.32 ( 33.48)	22.51 ( 31.14)	−.070
Depressive symptoms	Minimal	750(47.1)	666(46.9)	84(48.0)	2.586
	Mild	601(37.7)	531(37.4)	70(40.0)	
	Moderate	185(11.6)	171(12.1)	14(8.0)	
	Moderately Severe above	58(3.6)	51(3.6)	7(4.0)	
Stress symptoms		17.72 ( 4.41)	17.82 ( 4.43)	16.92 ( 4.16)	2.555^*^

^*^p < .05

### Factors associated with vitamin D level

The multivariable logistic regression model analysis revealed that the timing of the blood test was a significant factor influencing vitamin D levels (March–May; *p* < .01, June–August; *p* < .001, September–November; *p* < .01) as shown in Table 3.

**Table 3:** Odds ratio and 95% confidence interval for vitamin D levels from the multivariable logistic regression (*N* = 1594)

	Model 1		Model 2		Model 3		Model 4	
	OR	95% CI	OR	95% CI	OR	95% CI	OR	95% CI
Sampling month								
12–2	1		1		1		1	
3–5	2.228^**^	1.355,3.663	2.201^**^	1.328,3.648	2.257^**^	1.358,3.750	2.248^**^	1.350,3.743
6–8	2.170^***^	1.443,3.263	2.127^***^	1.407,3.216	2.103^***^	1.390,3.180	2.094^***^	1.383,3.173
9–11	2.227^**^	1.336,3.714	2.115^**^	1.251,3.575	2.169^**^	1.282,3.671	2.104^**^	1.240,3.571
Age (years)								
20–29			1		1		1	
30–39			2.383^***^	1.558,3.916	2.414^***^	1.519,3.834	2.383^***^	1.494,3.800
≥ 40			2.279^**^	1.415,4.597	2.296^**^	1.242,4.243	2.279^*^	1.221,4.254
Marital status								
Unmarried			1		1		1	
Married or other			.991	.677,1.449	.970	.660,1.426	1.019	.689,1.507
Education								
Diploma			1		1		1	
Bachelor			1.346	.827,2.190	1.374	.843,2.238	1.405	.860,2.295
Master’s or higher			1.284	.709,2.326	1.355	.745,2.463	1.324	.726,2.415
Shift work								
Yes					1		1	
No					1.193	.821,1.734	1.233	.847,1.797
Overtime hours (week)					.963	.927,1.001	.964	.927,1.002
BMI								
Underweight							1	
Normal							.775	.463,1.300
Overweight							.814	.462,1.435
Physical activity							1.001	.996,1.005
Depressive symptoms								
Minimal							1	
Mild							1.408	.967,2.051
Moderate							1.183	.604,2.317
Moderately severe above							2.457	.950,6.357
Stress symptoms							.942^**^	.900,.985
Nagelkerke R^2^	.024		.058		.064		.076	
Chi/df	18.879/3		46.855/8		51.737/10		61.644/17	

^*^p < .05, ^**^p < .01, ^***^p < .001.

Compared with participants who underwent blood tests in December–February, those who underwent blood tests in March–May (odds ratio [OR] = 2.248, confidence interval [CI] = 1.350, 3.743), June–August (OR = 2.094, CI = 1.383,3.173), and September–November (OR = 2.104, CI = 1.240, 3.571) presented with higher ORs for vitamin D levels greater than 20 ng/mL. Among the general characteristics, age was a significant factor influencing vitamin D levels (30–39; *p* < .001, 40 ≤; *p* = .010). Compared with the participants in their 20s, those in their 30s (OR = 2.383, CI = 1.494, 3.800) and 40s (OR = 2.279, CI = 1.221, 4.254) were more likely to have vitamin D levels over 20 ng/mL. Of the physical and psychological factors, stress symptoms (*p =* .009) were a significant factor influencing vitamin D levels: among participants with higher stress symptoms scores (OR = .942, CI = .900, .985), there were fewer participants with vitamin D levels over 20 ng/mL.

## Discussion

This study was conducted to determine the effects of vitamin D deficiency in women of childbearing age in South Korea. As indicated by the results, 89% of the participants were found to have low serum 25(OH) D concentrations, which is consistent with the results of a previous study which demonstrated that 88% of the women in their 20s and 30s were diagnosed with vitamin D deficiency [25]. As women (88.6%) have a higher incidence of low 25(OH) D levels than men (79.4%) among adults in their 20s [8], vitamin D deficiency should be recognized as one of the primary health concerns for women.

In this study, the sampling month, age, and stress were identified as factors influencing vitamin D levels. Vitamin D levels were lowest in participants who had their blood drawn between December and February. Given that vitamin D synthesis in the skin is affected by exposure to sunlight, individuals are likely to have relatively low concentrations of vitamin D during the winter months [26]. A previous study also reported that low sunlight exposure during the winter months was associated with vitamin D deficiency [27]. Concurrently, there is a high incidence of vitamin D deficiency among city residents as a result of working indoors and leading an indoor lifestyle [28]. In addition, Palacious and Gonzalez reported that women in the Middle East have lower serum 25(OH) D concentrations [29], owing to cultural influences on women’s preference for specific clothing (hijab) to avoid body exposure, resulting in the inhibition of vitamin D synthesis [30]. Alternatively, the incidence of inadequate 25(OH) D levels is low in beachfront residents [10]. Hence, the degree of sunlight exposure is an important factor affecting the level of vitamin D in the blood.

The participants with the lowest vitamin D levels were those in their 20s. This is consistent with the high vitamin D deficiency rates reported among Australian women aged 25 to 34 [5] and Chinese women in their 20s [27], which is important considering that low serum 25(OH) D concentration affects women’s childbirth, prenatal, and postpartum health [31]. A previous study reported that South Korean nurses work long hours, and 64.9% of nurses have low levels of physical activity [32]. Additionally, many women likely inhibit the synthesis of vitamin D in their skin by using sunscreen [33].

We also found that stress levels affect vitamin D deficiency in women of childbearing age, consistent with the results of a study by Gwon et al. which showed that perceived stress was a risk factor for low 25(OH) D levels in older women [12]. Abu-Samak et al. also reported that there was a weak positive association between vitamin D deficiency and elevated serum cortisol, although it is difficult to determine a direct link between low 25(OH) D levels and stress [34]. In a similar study, vitamin D levels decreased with the subjective perception of depression [15], while higher serum 25(OH) D levels showed a higher probability of positive self-perceived mental health [16]). These findings suggest that further studies on the link between vitamin D levels and psychological factors should be conducted.

Our results showed that factors that had no effect on vitamin D deficiency included level of education, shift work, physical activity level, and depression level, which is contrary to reports demonstrating that those with higher levels of education show a higher prevalence of 25(OH) D deficiency [28, 30]. This may be a consequence of the high level of awareness about vitamin D deficiency, its prevention, and treatment among nurses, who may therefore practice preventive management of vitamin D levels. Such a theory could be confirmed by comparing the differences between medical and non-medical professionals in the future.

Increased physical activity is known to play an important role in preventing low serum vitamin D levels; vitamin D synthesis increases with sun exposure during outdoor activity, which reduces sedentary activity and strengthens muscles [25, 28, 30]. In our study, the physical activity of the group with serum vitamin D levels ≥ 20 ng/mL was slightly higher than that of the group with vitamin D levels < 20 ng/mL; however, the difference was not statistically significant. Considering that our study participants were nurses, their physical activity primarily happens during work. Due to special circumstances, such as working long hours indoors or sleeping during daytime, their reported amount of physical activity cannot be counted as outdoor activity or as the amount of time exposed to the sun.

Our study has several limitations. First, as the study had a cross-sectional design, we cannot yet determine the causality of associations with diseases that may occur owing to vitamin D deficiency. Further longitudinal studies will need to be conducted on the prevalence of diseases associated with vitamin D deficiency. Second, bone densitometry measurement was not included in this study; thus, we could not assess the current bone health of participants. As bone densitometry is a basic tool for assessing bone health directly, simultaneous measurements of serum vitamin D levels and bone density are suggested. Third, there were gaps in time points of collecting data because physical activity was measured in M3 during November 2015–2017 and vitamin D level was measured in M5 during November 2016–2017. Physical activity will, thus, not reflect the levels of vitamin D directly.

Despite these limitations, our study has a major strength. Although we analyzed serum vitamin D levels of only 1594 samples, this is the first nationwide cohort study that included more than 10,000 registered nurses in South Korea. This study administers surveys to participants every year. Therefore, conducting further longitudinal studies to explore the association between the prevalence of diseases and general characteristic and clinical factors is promising. The study analyzed factors associated with vitamin D deficiency using self-reported data on job-related characteristics of South Korean nurses, thereby providing basic information for an intervention plan for female nurses of childbearing age.

## Conclusions

This study aimed to determine the current status of serum vitamin D levels and the risk factors for vitamin D deficiency among South Korean nurses of childbearing age. Our study results were consistent with the results of other cohort studies conducted in different parts of the world; hence, vitamin D deficiency appears to be a common health problem across the globe. However, this is the first nationwide study to investigate the current status of vitamin D levels of South Korean nurses of childbearing age. Therefore, our contribution to this field of study can be significant. Further studies should focus on investigating causal relationships between vitamin D deficiency and comprehensive risk factors such as sunlight exposure, lifestyle (i.e. skin coverage, physical exercise, and dietary patterns and supplements), adiposity, diseases, and demographics as well as long-term effects of untreated vitamin D deficiency.

## Data Availability

The datasets generated and analyzed during the current study are not publicly available. This is government data and time is required to enable data clearing and the establishment of guidelines. The Korea Centers for Disease Control and Prevention is planning on making the data available to the public in the future.

## Abbreviations

25[OH]D: 25-hydroxyvitamin D
CI: confidence interval
KNHS: Korean Nurses’ Health Study
MET: metabolic equivalent of task
OR: odds ratio
